# Comparing socio-economic inequalities in self-reported and undiagnosed hypertension among adults 45 years and over in India: what explains these inequalities?

**DOI:** 10.1186/s12939-023-01833-6

**Published:** 2023-02-02

**Authors:** Mrigesh Bhatia, Priyanka Dixit, Manish Kumar, Laxmi Kant Dwivedi

**Affiliations:** 1 London School of Economics, London, WC2A 2AE UK; 2 Tata Institute of Social Sciences, Mumbai, India; 3 Population Research Centre, Dharwad, India; 4 International Institute for Population Sciences, Mumbai, India

**Keywords:** Socio-economic inequalities, Self-reported hypertension, Undiagnosed hypertension, Decomposition, Older adults, India

## Abstract

**Background:**

Hypertension (HTN) is a leading cause of mortality and morbidity in developing countries. For India, the hidden burden of undiagnosed hypertension is a major concern. This study aims to assess and explain socio-economic inequalities among self-reported and undiagnosed hypertensives in India.

**Methods:**

The study utilized data from the Longitudinal Aging Study in India (LASI), a nationally-representative survey of more than 72,000 older adults. The study used funnel plots, multivariable logistic regression, concentration indices, and decomposition analysis to explain the socio-economic gap in the prevalence of self-reported and undiagnosed hypertension between the richest and the poorest groups.

**Results:**

The prevalence of self-reported and undiagnosed hypertension was 27.4 and 17.8% respectively. Monthly per capita consumption expenditure (MPCE) quintile was positively associated with self-reported hypertension but negatively associated with undiagnosed hypertension. The concentration index for self-reported hypertension was 0.133 (*p* < 0.001), whereas it was − 0.047 (*p* < 0.001) for undiagnosed hypertension. Over 50% of the inequalities in self-reported hypertension were explained by the differences in the distribution of the characteristics whereas inequalities remained unexplained for undiagnosed hypertension. Obesity and diabetes were key contributors to pro-rich inequality.

**Conclusions:**

Results imply that self-reported measures underestimate the true prevalence of hypertension and disproportionately affect the poorer MPCE groups. The prevalence of self-reported HTN was higher in the richest group, whereas socio-economic inequality in undiagnosed hypertension was significantly concentrated in the poorest group. As majority of the inequalities remain unexplained in case of undiagnosed hypertension, broader health systems issues including barriers to access to health care may be contributing to inequalities.

**Supplementary Information:**

The online version contains supplementary material available at 10.1186/s12939-023-01833-6.

## Background

Health inequalities are unjust and avoidable differences in health across population groups and are contrary to the principles of social justice [1]. Addressing them is important for both national governments and international organisations [2, 3]. Given the high prevalence of disability and death due to non-communicable diseases (NCDs), their chronic nature and its cost implications, reducing inequalities on account of NCDs assumes further importance. This is more so for a condition like hypertension (HTN) that can remain undetected for long and have significant implications in terms of cardiovascular morbidity and premature mortality.

Worldwide, the number of hypertensive adults has increased from 650 million to 1.28 billion in the last three decades [4]. HTN contributed to about 10.4 million deaths and 218 million disability adjusted life years (DALYs) in 2017 worldwide [5]. In recent years, the prevalence of HTN has declined in the high-income countries and most of the increase has occurred in low-income and middle-income regions [4]. India is no exception to this trend as HTN is a major public health problem in the country and accounts for about 7.9% of the total DALYs [6]. Compounding the problem is the hidden burden of HTN, that is, a significant proportion of Indians are unaware of their hypertensive status as they remain undiagnosed and untreated and are missed by the health system [7, 8]. Early diagnosis and prompt treatment is a crucial public health strategy to prevent avoidable morbidity and mortality due to HTN, which is a well-known risk factor for ailments of heart, brain, kidney and other diseases [9].

Understanding the socio-economic (SE) inequalities in the diagnosis and treatment of HTN within different groups of the population is essential for planning interventions and strategies for the prevention and control of HTN [10]. In recent years, there has been growing evidence to suggest the association between socio-economic status (SES) and HTN [7, 11–17]. Most of this body of research is based either on self-reported (SR) measures or on objective assessment of HTN. SR measures are commonly considered a reliable measure of the health of a population in the context of developed countries. However, given the extent of SE inequalities in the developing countries [18–20], the use of SR measures in such countries is likely to underestimate the true prevalence of HTN, especially in the lower socio-economic groups [21, 22]. For example, in a recent study by Bhatia et al. (2022), the prevalence of HTN based on SR measure, varied from 27.4 to 45.2%, respectively, among the poorest and the richest monthly per capita expenditure (MPCE) quintiles [7].

In order to meet the global and national target of 25% relative reduction in the prevalence of HTN by by 2025 [23, 24], an important first step is to quantify the hidden burden of undiagnosed HTN and identify undiagnosed and untreated individuals and SE groups that are missed out by the health system. Although there is a growing body of literature on SE inequalities in HTN, there are  hardly any studies that compare SE inequalities among the SR and undiagnosed hypertensives (those unaware of their hypertensive status but identified by objective measurement at the time of survey) in low- and middle-income countries. By estimating the concentration indices and decomposing the determinants, this study therefore aims to assess and explain the SE inequalities by comparing SR hypertensive adults with undiagnosed hypertensive adults over the age of 45 years in India.

## Methods

### Study design and participants

This study utilized data from the first wave of the Longitudinal Ageing Study in India (LASI) conducted between April 2017 and November 2018. LASI is a nationally-representative population-based longitudinal survey designed to explore social, health, and economic well-being of more than 72,000 individuals aged 45 years or older. The sample selection in LASI was based on a multistage stratified cluster sample design, wherein the data was collected through a face-to-face interview in each respondent’s household with the help of a computer-assisted personal interview (CAPI). The Indian Council of Medical Research (ICMR) extended the necessary guidance and ethical approval for conducting the LASI.

### Outcome variable

SR and undiagnosed HTN were the outcome variables in the study. For assessing SR HTN, the participants were asked: “Has any health professional ever told you that you have HTN or high blood pressure?” The participant was identified as hypertensive if s/he answered “Yes.” As part of biomarker measurements, the blood pressure was measured three times (with a one-minute gap) using an electronic monitor (Omron model HEM-7121). We took the average of the last two readings of systolic blood pressure (SBP) and diastolic blood pressure (DBP). Following the Joint National Committee (JNC) 7 guidelines, HTN was defined as an average SBP ≥ 140 mmHg or/and DBP ≥ 90 mmHg or current use of any antihypertensive medication [25]. Undiagnosed HTN was defined as an elevated clinic blood pressure (systolic/diastolic ≥140/90 mmHg) on objective assessment at the time of the survey without previous diagnosis or treatment by a health professional [26]. The overall estimate of HTN was the combination of SR and undiagnosed HTN.

### Explanatory variables

Data was also collected on other variables, including activities of daily living (ADL), body mass index (BMI), level of physical activity, use of cigarette smoking and smokeless tobacco, and alcohol consumption. Various household factors, including religion (Hindu, Muslim, Christian, and others), caste (Scheduled Tribe, Scheduled Caste, Other Backward Class, and others), and place of residence (rural and urban), were included in the analysis. We classified India into six broad geographical regions: North, Central, East, Northeast, West, and South. After standardizing the overall expenditure (food and non-food) to a 30-day reference period, LASI computed the MPCE, which is used as a summary measure of consumption. Based on the MPCE, individuals were classified into poorest, poorer, middle, richer, and richest quintiles. Finally, we included various demographic and other background variables such as sex (male and female), age (45–54, 55–64, 65–74, and 75+ years), level of education (no education, primary, secondary, and higher), marital status (currently married, widowed and divorced/ separated/ deserted), and working status (never worked, currently working, and not currently working) in the analysis.

### Statistical analyses

This study had two outcomes variables: SR HTN (yes and no) and undiagnosed HTN (yes and no). The proportion test was used to assess if there was any significant difference between the prevalence of SR HTN and undiagnosed HTN across various background characteristics. We constructed funnel plots to observe the variation in the prevalence of SR (aware) and undiagnosed HTN between the poorest and richest categories across states of India. The national average of SR and undiagnosed HTN (indicated by a solid line parallel to the x-axis) was used as the baseline reference. The 95 and 99% confidence bands were also created on the funnel plots. Furthermore, we have drawn scatter plots where each data point indicates the state’s position regarding SR and undiagnosed HTN and the difference in the poorest (bottom 20%) and richest category (top 20%). Multivariable logistic regression was used to assess the association of SR and undiagnosed HTN after controlling for individual factors (age, education, marital status, and working status), morbidities (diabetes, stroke, arthritis, and difficulties with Activities of daily living (ADLs) and Instrumental activities of daily living (IADLs), lifestyle factors (moderate and vigorous activities, alcohol use, smoking and chewing tobacco status) and household factors (MPCE quintile, caste, religion, region, and residence).

Concentration indices (CIs) were calculated to measure the extent of the MPCE quintile-based inequalities in the SR, and undiagnosed HTN. In addition, the socio-economic gap in the prevalence of SR and undiagnosed HTN between the richest and the poorest groups was decomposed into the contributing individual, lifestyle, and household determinants. The observed difference in the prevalence of SR HTN between the two groups (richest and poorest) was additively decomposed into the endowment component (characteristics) and the coefficient component (effect of characteristics) [27]. All the analysis was carried out using the Stata 15.1 software.

## Results

Table S1 shows the characteristics of the study participants (n = 65,562) aged 45 years and above in terms of individuals factors, co-morbidities and anthropometric status, lifestyle factors, and household characteristics. Table 1 presents the prevalence of SR, undiagnosed, and overall HTN according to various individuals, biological, lifestyle, and household characteristics. At the all-India level, the prevalence of SR, undiagnosed, and overall HTN was 27.4% (95% Confidence Interval (CI): 26.5, 28.3), 17.8% (95% CI: 17.1, 18.5), and 45.2% (95% CI: 44.3, 46.1) respectively. The results from the proportion test indicate that there is a significance difference in the prevalence of SR HTN and undiagnosed HTN overall and across some background characteristics including age-groups, gender, educational level, marital status, morbidities, religion, caste, and place of residence. Almost four in ten individuals were undiagnosed hypertensives. The prevalence of SR and overall HTN increased with age and education, while that of undiagnosed HTN was uniform across age groups and educational levels. An important finding was that SR and overall HTN increased with increasing MPCE quintiles, while undiagnosed HNT decreased with increasing MPCE quintiles. For example, SR HTN was 36% in the highest MPCE quintile as compared to only 21% in the lowest MPCE quintile. Almost one in every two adults was an undiagnosed hypertensive in the lowest MCPE quintile as compared to less than one in three adults in the highest MPCE group. Those with co-morbidities like diabetes, stroke, arthritis, and obesity were more aware about their hypertensive status. Rural residents and those belonging to the Central and Western regions had higher proportions of undiagnosed HTN.

**Table 1:** Prevalence of self-reported, undiagnosed, and overall HT among older adults in India, 2017–18

	Self-reported HT	Undiagnosed HT	Overall HT
	% (95% CI)	% (95% CI)	% (95% CI)
**Individual factors**			
**Age groups**			
45–54	20.5 (19.0,22.1)	16.4 (14.8,18.0)	36.9 (35.1,38.7)
55–64	27.6 (26.3,28.8)	17.9 (17.0,18.8)	45.5 (44.1,46.8)
65–74	34.1 (32.1,36.0)	19.3 (18.1,20.5)	53.3 (51.6,55.1)
75+	34.2 (31.7,36.6)	18.6 (16.8,20.4)	52.7 (49.9,55.6)
**Sex**			
Men	23.2 (22.1,24.4)	19.2 (18.1,20.2)	42.4 (41.1,43.7)
Women	30.9 (29.7,32.2)	16.6 (15.7,17.5)	47.5 (46.3,48.8)
**Education level**			
No education	24.3 (23.4,25.3)	18.2 (17.4,18.9)	42.5 (41.5,43.5)
Primary	29 (27.6,30.3)	18.1 (17.1,19.2)	47.1 (45.7,48.5)
Secondary	31.6 (28.7,34.5)	16.1 (14.8,17.3)	47.7 (45.0,50.3)
Higher	32.4 (28.2,36.5)	17.9 (13.0,22.7)	50.2 (45.4,55.1)
**Working Status**			
Never worked	35.5 (33.4,37.6)	15.1 (13.5,16.8)	50.7 (48.6,52.7)
Currently working	18.8 (17.8,19.9)	19.3 (18.3,20.4)	38.2 (37.0,39.4)
Not currently working	34 (32.4,35.6)	17.7 (16.7,18.7)	51.7 (50.1,53.3)
**Marital Status**			
Currently married	25.4 (24.4,26.3)	17.4 (16.5,18.2)	42.7 (41.7,43.8)
Widowed	34.5 (32.5,36.5)	19.1 (17.9,20.2)	53.6 (51.8,55.4)
D/S/D/Others^a^	20.8 (16.7,24.9)	17.7 (14.1,21.3)	38.5 (32.3,44.8)
**Morbidities**			
**Diabetes**			
No	22.4 (21.8,23.0)	19 (18.2,19.7)	41.4 (40.5,42.2)
Yes	63.8 (60.8,66.8)	10.2 (9.0,11.4)	74 (71.6,76.4)
**Stroke**			
No	26.9 (26.0,27.7)	18 (17.3,18.7)	44.9 (44.0,45.8)
Yes	60.8 (56.3,65.3)	9.6 (7.2,12.0)	70.4 (66.4,74.5)
**Arthritis**			
No	26.2 (25.4,27.0)	18.3 (17.6,19.1)	44.5 (43.6,45.4)
Yes	40.6 (36.2,45.1)	13.2 (11.6,14.8)	53.9 (49.7,58.0)
**Difficulty in ADL**^**b**^			
No	25.8 (24.9,26.7)	18 (17.2,18.8)	43.8 (42.8,44.7)
Yes	36.1 (33.8,38.3)	17.5 (16.0,19.0)	53.6 (51.5,55.6)
**Difficulty in IADL**^**c**^			
No	24.8 (23.8,25.7)	18.1 (17.2,19.1)	42.9 (41.8,44.0)
Yes	32.1 (30.5,33.7)	17.5 (16.6,18.5)	49.6 (48.2,51.1)
**BMI categories**			
Normal	24.2 (23.4,25.0)	20.4 (19.5,21.2)	44.5 (43.5,45.5)
Underweight	15.5 (14.4,16.6)	17.1 (16.0,18.2)	32.6 (31.1,34.1)
Overweight/obese	41.6 (39.3,43.8)	20.9 (18.7,23.1)	62.4 (60.5,64.4)
**Lifestyle factors**			
**Moderate activities**			
Inactive	29.9 (28.9,30.9)	18 (17.2,18.9)	48 (46.9,49.1)
Active	26 (24.8,27.3)	17.9 (16.9,18.9)	44 (42.7,45.2)
**Vigorous activities**			
Inactive	30.8 (29.7,31.8)	17.5 (16.7,18.4)	48.3 (47.3,49.3)
Active	20.5 (19.0,22.0)	18.9 (17.5,20.3)	39.4 (37.7,41.1)
**Smoking status**			
Never	28.7 (27.7,29.7)	17.9 (17.1,18.7)	46.6 (45.6,47.6)
Former	31.7 (28.1,35.4)	16.5 (14.3,18.7)	48.2 (44.9,51.6)
Current	18.5 (17.4,19.7)	19 (17.6,20.4)	37.5 (35.9,39.1)
**Chewing tobacco status**			
Never	29 (27.9,30.1)	17.4 (16.5,18.3)	46.4 (45.3,47.5)
Former	33.4 (28.6,38.3)	18 (14.7,21.2)	51.4 (46.6,56.1)
Current	21.2 (20.1,22.4)	20.1 (19.0,21.2)	41.3 (40.0,42.7)
**Alcohol use**			
No	28.6 (27.6,29.6)	17.1 (16.3,17.8)	45.7 (44.6,46.7)
Yes	21.3 (19.9,22.6)	23.1 (21.8,24.4)	44.4 (42.8,45.9)
**Household factors**			
**MPCE quintile**			
Poorest	21 (19.8,22.2)	19.6 (18.4,20.8)	40.6 (39.1,42.0)
Poorer	24.5 (23.2,25.8)	18 (17.0,19.0)	42.5 (41.1,43.9)
Middle	26.4 (24.8,27.9)	18.5 (16.6,20.5)	44.9 (42.8,46.9)
Richer	30.7 (28.6,32.8)	16.4 (15.1,17.7)	47.1 (45.0,49.2)
Richest	35.9 (32.9,39.0)	16.2 (13.9,18.4)	52.1 (49.2,54.9)
**Religion**			
Hindu	26.1 (25.3,27.0)	17.9 (17.1,18.7)	44 (43.1,45.0)
Muslim	34.1 (30.4,37.9)	16.7 (14.7,18.7)	50.8 (47.3,54.2)
Christian	27 (22.1,31.8)	17.8 (14.7,20.9)	44.7 (38.0,51.5)
Others^d^	35.3 (32.7,37.9)	19 (16.8,21.2)	54.3 (51.5,57.2)
**Caste**			
Scheduled Caste	24.4 (23.1,25.8)	17.6 (16.5,18.7)	42 (40.5,43.5)
Scheduled Tribe	15.3 (14.0,16.6)	21.9 (20.4,23.3)	37.2 (35.3,39.0)
OBC^e^	27.7 (26.1,29.4)	17.9 (16.6,19.3)	45.7 (44.0,47.4)
Others	32.4 (31.3,33.6)	16.4 (15.5,17.2)	48.8 (47.6,50.0)
**Place of Residence**			
Rural	22.7 (22.1,23.3)	18.2 (17.7,18.8)	40.9 (40.2,41.6)
Urban	37.7 (35.3,40.0)	16.8 (14.9,18.6)	54.5 (52.2,56.8)
**Region**			
North	33.9 (32.7,35.1)	16 (15.1,16.9)	49.9 (48.7,51.2)
Central	19.7 (18.6,20.8)	17.2 (16.1,18.4)	36.9 (35.5,38.3)
East	25.6 (24.5,26.8)	16.8 (15.8,17.8)	42.4 (41.1,43.7)
Northeast	29.5 (27.9,31.0)	18.3 (17.0,19.5)	47.7 (46.1,49.4)
West	28 (26.5,29.4)	20.4 (19.0,21.7)	48.3 (46.7,49.9)
South	31.7 (28.7,34.7)	18.3 (15.9,20.7)	50 (47.0,53.0)
**India**	27.4 (26.5,28.3)	17.8 (17.1,18.5)	45.2 (44.3,46.1)

*Note*. *HT* Hypertension; Prevalence of Self-reported HT, undiagnosed HT, and overall HT was weighted

^a^divorced, separated, and deserted

^b^Activities of daily living

^c^Instrumental Activities of Daily Living (IADL)

^d^includes Sikh, Buddhist/neo-Buddhist, Jain, Parsi/Zoroastrian and others; and

^e^Other Backward Classes

Table 2 shows the logistic regression estimates for SR and undiagnosed HTN among Indian older adults. The results suggest that higher age and higher education level were significantly positively associated with SR HTN. Being overweight/obese, having co-morbidities like diabetes, stroke, and arthritis, and having functional limitations in ADLs and IADLs increased the odds of awareness about one’s HTN status. An important observation was that MPCE quintile was positively associated with SR HTN but negatively associated with undiagnosed HTN, indicating that older adults in the higher MPCE category were more aware of their HTN status, whereas those belonging to lower MPCE groups tended to be undiagnosed.

**Table 2:** Logistic regression results for self-reported and undiagnosed HT among older adults, India, LASI, 2017–18

	Self-reported HT						Undiagnosed HT					
	OR (Overall)	95% CI	OR (Men)	95% CI	OR (Women)	95% CI	OR (Overall)	95% CI	OR (Men)	95% CI	OR (Women)	95% CI
**Individual factors**												
**Age groups**												
45–54	Ref.		Ref.		Ref.		Ref.		Ref.		Ref.	
55–64	1.39***	(1.32,1.46)	1.41***	(1.30,1.53)	1.41***	(1.32,1.50)	1.30***	(1.23,1.37)	1.16***	(1.07,1.24)	1.42***	(1.32,1.53)
65–74	1.76***	(1.66,1.87)	1.88***	(1.71,2.07)	1.81***	(1.68,1.96)	1.43***	(1.35,1.52)	1.18***	(1.08,1.29)	1.60***	(1.46,1.75)
75+	1.80***	(1.66,1.95)	2.07***	(1.82,2.34)	1.85***	(1.66,2.07)	1.64***	(1.51,1.78)	1.31***	(1.15,1.48)	1.81***	(1.60,2.04)
**Education level**												
No education	Ref.		Ref.		Ref.		Ref.		Ref.		Ref.	
Primary	1.13***	(1.07,1.19)	1.18***	(1.09,1.29)	1.25***	(1.16,1.34)	1	(0.95,1.06)	1.02	(0.94,1.10)	0.91*	(0.84,0.99)
Secondary	1.06	(0.99,1.12)	1.25***	(1.14,1.37)	1.09	(1.00,1.19)	1.07*	(1.01,1.14)	1.03	(0.95,1.12)	0.94	(0.84,1.05)
Higher	1.11*	(1.02,1.20)	1.42***	(1.27,1.59)	0.98	(0.86,1.11)	1.05	(0.97,1.14)	0.97	(0.87,1.08)	0.91	(0.78,1.07)
**Working Status**												
Never worked	Ref.		Ref.		Ref.		Ref.		Ref.		Ref.	
Currently working	0.69***	(0.66,0.73)	0.81*	(0.69,0.95)	0.79***	(0.74,0.85)	1.18***	(1.11,1.25)	0.99	(0.85,1.17)	0.98	(0.91,1.07)
Not currently working	0.97	(0.92,1.02)	1.13	(0.96,1.33)	1.05	(0.98,1.12)	1.09**	(1.02,1.16)	0.94	(0.80,1.10)	1	(0.93,1.09)
**Marital Status**												
Currently married	Ref.		Ref.		Ref.		Ref.		Ref.		Ref.	
Widowed	1.29***	(1.23,1.36)	0.97	(0.87,1.08)	1.30***	(1.23,1.39)	1.13***	(1.07,1.19)	1.15**	(1.03,1.27)	1.18***	(1.10,1.26)
D/S/D/Others^a^	1.00	(0.89,1.12)	0.85	(0.70,1.02)	1.1	(0.95,1.28)	1.16*	(1.03,1.30)	1.16	(0.99,1.37)	1.20*	(1.02,1.40)
**Morbidities**												
**Diabetes**												
No	Ref.		Ref.		Ref.		Ref.		Ref.		Ref.	
Yes	3.51***	(3.32,3.72)	3.61***	(3.33,3.92)	3.46***	(3.20,3.74)	0.50***	(0.47,0.54)	0.50***	(0.45,0.55)	0.50***	(0.45,0.56)
**Stroke**												
No	Ref.		Ref.		Ref.		Ref.		Ref.		Ref.	
Yes	3.32***	(2.85,3.87)	3.62***	(2.97,4.40)	3.03***	(2.37,3.88)	0.52***	(0.42,0.64)	0.48***	(0.37,0.63)	0.55***	(0.39,0.78)
**Arthritis**												
No	Ref.		Ref.		Ref.		Ref.		Ref.		Ref.	
Yes	1.39***	(1.30,1.49)	1.32***	(1.17,1.48)	1.42***	(1.30,1.54)	0.73***	(0.67,0.80)	0.76***	(0.66,0.86)	0.74***	(0.66,0.82)
**Difficulty in ADL**^b^												
No	Ref.		Ref.		Ref.		Ref.		Ref.		Ref.	
Yes	1.20***	(1.13,1.27)	1.27***	(1.15,1.41)	1.16***	(1.07,1.25)	0.93*	(0.87,1.00)	0.93	(0.84,1.04)	0.93	(0.85,1.02)
**Difficulty in IADL**^**c**^												
No	Ref.		Ref.		Ref.		Ref.		Ref.		Ref.	
Yes	1.17***	(1.11,1.22)	1.15***	(1.06,1.25)	1.13***	(1.06,1.20)	0.96	(0.91,1.01)	0.97	(0.90,1.05)	0.99	(0.92,1.06)
**BMI categories**												
Normal	Ref.		Ref.		Ref.		Ref.		Ref.		Ref.	
Underweight	0.64***	(0.60,0.68)	0.63***	(0.58,0.70)	0.64***	(0.59,0.70)	0.77***	(0.73,0.82)	0.71***	(0.65,0.77)	0.85***	(0.78,0.92)
Overweight/obese	1.77***	(1.69,1.85)	1.68***	(1.56,1.80)	1.79***	(1.69,1.90)	1.16***	(1.11,1.22)	1.26***	(1.18,1.36)	1.14***	(1.07,1.23)
**Lifestyle factors**												
**Moderate activities**												
Inactive	Ref.		Ref.		Ref.		Ref.		Ref.		Ref.	
Active	0.97	(0.92,1.01)	0.95	(0.89,1.02)	0.90***	(0.85,0.95)	1.01	(0.96,1.05)	1.06	(1.00,1.14)	1.10**	(1.02,1.18)
**Vigorous activities**												
Inactive	Ref.		Ref.		Ref.		Ref.		Ref.		Ref.	
Active	0.91***	(0.86,0.96)	0.90**	(0.83,0.96)	0.97	(0.90,1.04)	1.04	(0.99,1.09)	0.97	(0.91,1.04)	1.08	(1.00,1.16)
**Smoking status**												
Never	1	(1.00,1.00)	1	(1.00,1.00)	1	(1.00,1.00)	Ref.		Ref.		Ref.	
Former	1.04	(0.95,1.15)	1.06	(0.95,1.18)	1.25	(0.99,1.58)	0.85**	(0.77,0.94)	0.84**	(0.75,0.95)	0.65**	(0.48,0.88)
Current	0.85***	(0.79,0.91)	0.91*	(0.84,0.98)	0.99	(0.85,1.15)	0.96	(0.90,1.03)	0.93	(0.86,1.00)	0.79**	(0.66,0.94)
**Chewing tobacco status**												
Never	Ref.		Ref.		Ref.		Ref.		Ref.		Ref.	
Former	1.05	(0.92,1.19)	1.01	(0.86,1.20)	1.13	(0.92,1.40)	0.97	(0.85,1.11)	0.95	(0.80,1.12)	0.97	(0.77,1.22)
Current	0.93**	(0.88,0.98)	0.89**	(0.82,0.97)	1.03	(0.95,1.12)	1.05	(0.99,1.11)	1.02	(0.95,1.10)	1.08	(0.99,1.17)
**Alcohol use**												
No	Ref.		Ref.		Ref.		Ref.		Ref.		Ref.	
Yes	0.95	(0.89,1.01)	1.06	(0.99,1.14)	0.86	(0.74,1.01)	1.45***	(1.37,1.54)	1.33***	(1.24,1.42)	1.39***	(1.22,1.60)
**Household factors**												
**MPCE quintile**												
Poorest	Ref.		Ref.		Ref.		Ref.		Ref.		Ref.	
Poorer	1.15***	(1.08,1.23)	1.08	(0.98,1.20)	1.19***	(1.09,1.29)	0.91**	(0.85,0.97)	0.94	(0.86,1.03)	0.89*	(0.82,0.98)
Middle	1.25***	(1.17,1.34)	1.16**	(1.04,1.28)	1.30***	(1.20,1.42)	0.90**	(0.84,0.96)	0.95	(0.87,1.04)	0.87**	(0.80,0.96)
Richer	1.37***	(1.28,1.46)	1.30***	(1.17,1.44)	1.39***	(1.28,1.52)	0.85***	(0.80,0.91)	0.89*	(0.81,0.98)	0.84***	(0.76,0.92)
Richest	1.42***	(1.33,1.52)	1.36***	(1.23,1.51)	1.43***	(1.30,1.56)	0.81***	(0.75,0.86)	0.84***	(0.76,0.93)	0.81***	(0.73,0.89)
**Religion**												
Hindu	Ref.		Ref.		Ref.		Ref.		Ref.		Ref.	
Muslim	1.23***	(1.15,1.31)	1.12*	(1.01,1.24)	1.38***	(1.27,1.50)	1.04	(0.97,1.12)	0.99	(0.90,1.10)	1.02	(0.92,1.12)
Christian	1.03	(0.94,1.12)	1	(0.87,1.15)	1.05	(0.94,1.18)	0.96	(0.88,1.05)	0.92	(0.81,1.04)	1.01	(0.90,1.14)
Others^d^	1.26***	(1.15,1.39)	1.27***	(1.10,1.47)	1.34***	(1.18,1.52)	1.07	(0.97,1.18)	1.1	(0.96,1.27)	0.99	(0.85,1.15)
**Caste**												
Scheduled Caste	Ref.		Ref.		Ref.		Ref.		Ref.		Ref.	
Scheduled Tribe	0.76***	(0.70,0.82)	0.78***	(0.69,0.89)	0.74***	(0.66,0.82)	1.38***	(1.28,1.49)	1.40***	(1.26,1.56)	1.39***	(1.25,1.55)
OBC^e^	0.99	(0.93,1.05)	0.98	(0.89,1.07)	1.00	(0.92,1.08)	0.94*	(0.88,1.00)	1	(0.92,1.09)	0.88**	(0.81,0.96)
Others	1.04	(0.97,1.11)	1.07	(0.96,1.18)	1.02	(0.93,1.11)	0.98	(0.91,1.05)	0.93	(0.85,1.03)	1.03	(0.93,1.14)
**Place of Residence**												
Rural	Ref.		Ref.		Ref.		Ref.		Ref.		Ref.	
Urban	1.19***	(1.14,1.25)	1.19***	(1.11,1.28)	1.18***	(1.11,1.26)	0.99	(0.95,1.04)	1.08*	(1.01,1.15)	0.92*	(0.85,0.99)
**Region**												
North	Ref.		Ref.		Ref.		Ref.		Ref.		Ref.	
Central	0.67***	(0.62,0.72)	0.69***	(0.61,0.78)	0.66***	(0.60,0.73)	1.08*	(1.00,1.17)	0.88*	(0.79,0.99)	1.35***	(1.20,1.51)
East	0.84***	(0.79,0.91)	0.92	(0.82,1.03)	0.81***	(0.74,0.88)	1.02	(0.95,1.10)	0.80***	(0.72,0.89)	1.29***	(1.16,1.44)
Northeast	1.00	(0.92,1.10)	1.10	(0.96,1.26)	0.87*	(0.78,0.99)	1.07	(0.98,1.18)	1.02	(0.90,1.15)	1.18*	(1.03,1.35)
West	0.77***	(0.71,0.83)	0.83**	(0.74,0.93)	0.71***	(0.64,0.78)	1.19***	(1.10,1.29)	1.07	(0.96,1.20)	1.36***	(1.21,1.53)
South	0.84***	(0.79,0.90)	0.96	(0.87,1.06)	0.76***	(0.70,0.83)	1.27***	(1.18,1.36)	1.09	(0.99,1.21)	1.50***	(1.35,1.66)

*Note.*^a^divorced, separated, and deserted

^b^Activities of daily living

^c^Instrumental Activities of Daily Living (IADL)

^d^includes Sikh, Buddhist/neo-Buddhist, Jain, Parsi/Zoroastrian and others; and

^e^Other Backward Classes, HT-hypertension

*p < 0.05, **p < 0.01, ***p < 0.001

**Fig. 1:**
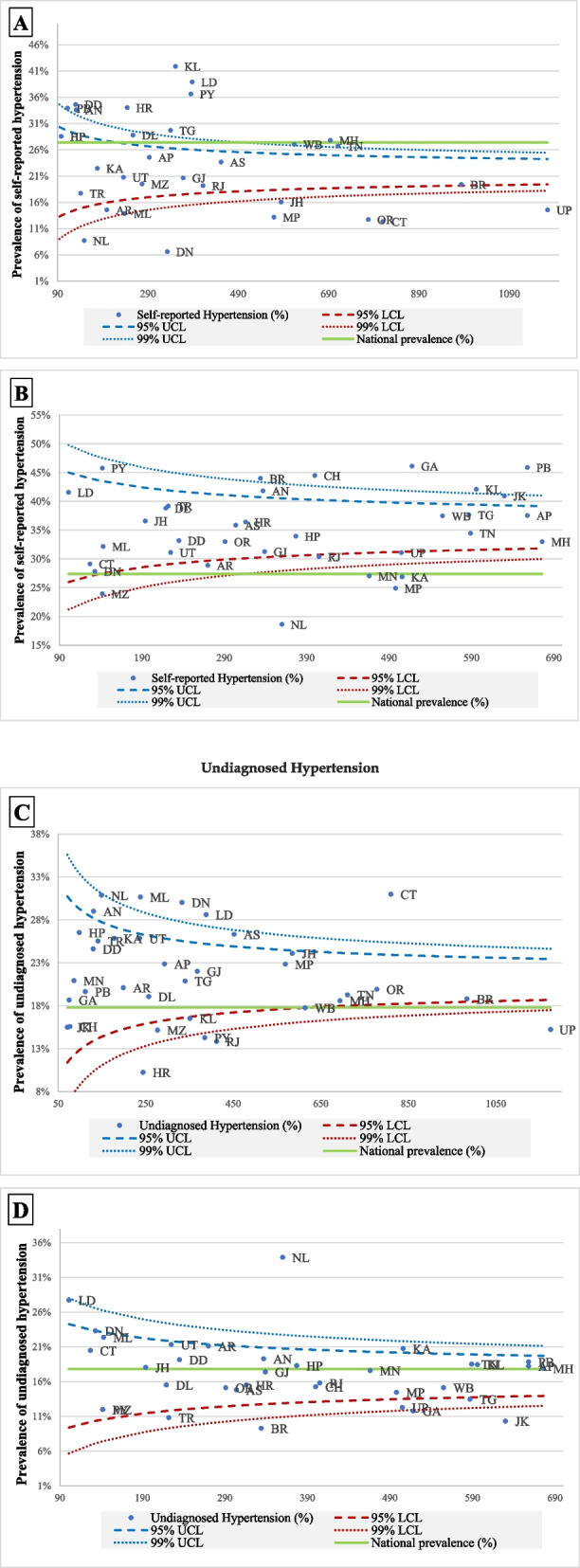
**A**-**D** Funnel plots for self-reported and undiagnosed hypertension among older adults, LASI, 2017–18; **A** Self-reported HTN in poorest group **B** Self-reported HTN in richest group **C** Undiagnosed HTN in poorest group **D** Undiagnosed HTN in richest group

Figure 1 (A-D) present the funnel plots for the SR and undiagnosed HTN for the poorest and richest categories. The ‘green’ line represents the national prevalence of the SR and undiagnosed HTN, and the states outside the 99% CI are outliers. High prevalence of SR HTN in the richest category is observed with large number of states above the national prevalence among the richest as compared with the poorest quintile (Fig. 1A, B). The opposite is observed for undiagnosed HTN. Similarly, Figure S1 presents the differential in prevalence of HTN between the richest and the poorest quintile for SR and undiagnosed HTN. High pro rich differentials with huge variation among the MCPE groups is observed for SR HTN whereas pro poor differential is observed in case of undiagnosed HTN. Fig. S1 (B) shows states like Chhattisgarh, Meghalaya, Jharkhand, Gujarat, and Uttarakhand were the worst-performing states both in terms of the high prevalence of undiagnosed HTN and high differentials between rich and poor. On the other hand, states like Mizoram, Haryana, West Bengal, and Rajasthan have performed well and indicate better health systems response, leading to a low prevalence of undiagnosed HTN and low differentials between two extreme wealth groups

It can be observed from the Table S2 that the concentration index (CI) for SR HTN was 0.133 (*p* < 0.001), indicating pro-rich MPCE-based inequalities, whereas for undiagnosed HTN, it was − 0.047 (*p* < 0.001), indicating pro-poor inequality in the prevalence of undiagnosed HTN. The concentration index of undiagnosed HTN was found to be negative for all the categories of selected covariates as opposed to SR HTN which clearly indicates that prevalence of undiagnosed HTN was more concentrated among the poorest category.

Finally, Table 3 shows the overall decomposition results for the covariates contributing to the inequality in propensity to self-report HTN between the bottom 20% poorest and the top 20% richest MPCE groups. The logit decomposition reveals that nearly 52% of the inequalities in HTN status were explained by the differences in the distribution of characteristics, namely age, sex, education, working status, morbidity and BMI status, religion, caste, and place and state of residence. There would have been about 18 to 19% less inequality in the propensity to self-report HTN between the poorest and the richest if diabetes could be cured and overweight/obese individuals became normal. Each underweight and scheduled tribe individuals would result in 6% reduction in the inequality in the prevalence of HTN between the two wealth groups if the distribution was similar to that of individuals with a normal BMI and scheduled caste individuals respectively. In terms of the propensity component, those who experienced difficulty with ADLs caused the inequality to increase by 9% compared to those who did not experience difficulty with ADLs, while moderately active people contributed 21% more relative to inactive people to the increase in the inequality. The states of Uttar Pradesh and Bihar caused the inequality in the propensity to report HTN between the poorest and the richest groups to decline by nearly 13–15% in comparison to Tamil Nadu. In case of SR HTN selected covariates characteristics clearly explains the major inequality, however in case of diagnosed HTN few covariates coefficients like age, gender, marital status and place of state are contributing to some extent but a major portion of inequality has remained unexplained.

**Table 3:** Logit decomposition of contribution of various factors in prevalence of self-reported and undiagnosed hypertension among bottom 20% poorest and top 20% richest population, India, LASI, 2017–18

	**Self-reported Hypertension**						**Undiagnosed Hypertension**					
	**Coefficient**	**SE**	**Percent**				**Coefficient**	**SE**	**Percent**			
Due to Difference in Characteristics (E)	0.0711***	0.0087	51.9%				0.00245	0.0115	−8.1%			
Due to Difference in Coefficients (C)	0.0660***	0.0113	48.1%				−0.0328***	0.0120	108.1%			
Overall difference (R)	0.1371***	0.0104					−0.03035	0.0123				
**Covariates**	**Due to Difference in Characteristics (E)**			**Due to Difference in Coefficients (C)**			**Due to Difference in Characteristics (E)**			**Due to Difference in Coefficients (C)**		
	**Coefficient**	**SE**	**Percent**	**Coefficient**	**SE**	**Percent**	**Coefficient**	**SE**	**Percent**	**Coefficient**	**SE**	**Percent**
**Age groups**												
45–54												
55–64	0.00031***	0.000	0.2%	0.00355	0.008	2.6%	−0.00012	0.000	0.4%	− 0.02112**	0.006	69.6%
65–74	− 0.00171***	0.000	−1.2%	− 0.00579	0.006	−4.2%	− 0.00043	0.000	1.4%	−0.0068	0.006	22.4%
75+	−0.00229***	0.001	−1.7%	−0.00145	0.004	−1.1%	−0.00070	0.001	2.3%	−0.0024	0.004	7.9%
**Gender**												
Male												
Female	−0.00071*	0.000	−0.5%	0.00180	0.015	1.3%	0.00106	0.001	−3.5%	−0.03724**	0.013	122.7%
**Education level**												
No education												
Primary	0.00066***	0.000	0.5%	0.00501	0.005	3.7%	−0.00028	0.000	0.9%	−0.0051	0.004	17.0%
Secondary	0.00362***	0.002	2.6%	− 0.00296	0.004	−2.2%	−0.00292	0.004	9.6%	0.0013	0.003	−4.3%
Higher	0.00830	0.005	6.1%	0.00164	0.002	1.2%	−0.00453	0.005	14.9%	−0.0019	0.002	6.1%
**Working Status**												
Never worked												
Currently working	0.00436***	0.001	3.2%	−0.01894	0.014	−13.8%	− 0.00204	0.002	6.7%	−0.0012	0.012	4.1%
Not currently working	0.00031	0.000	0.2%	−0.00456	0.007	−3.3%	−0.00015	0.000	0.5%	− 0.0090	0.007	29.7%
**Marital Status**												
Currently married												
Widowed	−0.00179	0.001	−1.3%	0.00069	0.006	0.5%	−0.00212	0.002	7.0%	−0.0025	0.005	8.2%
D/S/D/Others^a^	−0.00011	0.000	0.1%	0.00165	0.001	1.2%	0.00011	0.000	−0.4%	− 0.00301**	0.001	9.9%
**Morbidities**												
**Diabetes**												
No												
Yes	0.02433***	0.002	17.7%	0.00039	0.002	0.3%	−0.01045	0.012	34.4%	− 0.0005	0.003	1.7%
**Stroke**												
No												
Yes	0.00274***	0.001	2.0%	0.00059	0.001	0.4%	−0.00186	0.002	6.1%	−0.0013	0.001	4.2%
**Arthritis**												
No												
Yes	0.00381***	0.001	2.8%	0.00476	0.002	3.5%	−0.00391	0.005	12.9%	−0.0022	0.003	7.1%
**Difficulty in ADL**^**b**^												
No												
Yes	0.00018	0.000	0.1%	−0.01273**	0.004	−9.3%	−0.00049	0.001	1.6%	0.0066	0.004	−21.8%
**Difficulty in IADL**^**c**^												
No												
Yes	−0.00282*	0.001	−2.1%	0.00876	0.008	6.4%	0.00031	0.001	−1.0%	− 0.0057	0.007	18.6%
**BMI categories**												
Normal												
Underweight	0.00778*	0.004	5.7%	0.00585	0.007	4.3%	0.00976	0.009	−32.2%	−0.0046	0.006	15.2%
Overweight/obese	0.02563***	0.004	18.7%	0.00694	0.004	5.1%	0.00993	0.013	−32.7%	0.0000	0.004	0.0%
**Lifestyle factors**												
**Moderate activities**												
Inactive												
Active	0.00032*	0.000	0.2%	−0.02912*	0.014	−21.2%	− 0.00007	0.000	0.2%	0.0097	0.011	−31.9%
**Vigorous activities**												
Inactive												
Active	− 0.00011	0.000	0.1%	0.00617	0.008	4.5%	−0.00007	0.000	0.2%	0.0020	0.007	−6.5%
**Smoking Status**												
Never												
Former	−0.00011	0.000	0.1%	− 0.00249	0.001	−1.8%	0.00000	0.000	0.0%	0.0022	0.001	−7.1%
Current	0.00018	0.000	0.1%	−0.00148	0.004	− 1.1%	0.00018	0.000	−0.6%	− 0.0016	0.003	5.3%
**Chewing tobacco Status**												
Never												
Former	0.00000	0.000	0.0%	−0.00114	0.002	− 0.8%	− 0.00004	0.000	0.1%	0.0016	0.001	−5.2%
Current	0.00324	0.002	2.4%	−0.00235	0.005	−1.7%	−0.00249	0.002	8.2%	0.0050	0.005	−16.5%
**Alcohol use**												
No												
Yes	−0.00056	0.001	−0.4%	0.00214	0.004	1.6%	−0.00078	0.001	2.6%	−0.0016	0.003	5.1%
**Household factors**												
**Religion**												
Hindu												
Muslim	−0.00198*	0.001	−1.4%	0.00199	0.004	1.5%	−0.00002	0.001	0.0%	−0.0015	0.004	4.8%
Christian	0.00003	0.000	0.0%	−0.00130	0.001	−1.0%	0.00012	0.000	−0.4%	−0.0011	0.001	3.6%
Others^d^	0.00176	0.001	1.3%	0.00105	0.001	0.8%	−0.00003	0.001	0.1%	0.0004	0.001	−1.3%
**Caste**												
Scheduled Caste												
Scheduled Tribe	0.00858*	0.004	6.3%	−0.00073	0.006	−0.5%	0.00077	0.003	−2.5%	−0.0041	0.005	13.4%
OBC^e^	−0.00007	0.000	0.0%	−0.01530	0.012	−11.2%	−0.00001	0.000	0.0%	−0.0102	0.011	33.6%
Others	−0.00335	0.004	−2.4%	−0.00296	0.004	−2.2%	−0.00202	0.004	6.7%	−0.0015	0.004	4.9%
**Place of Residence**												
Rural												
Urban	0.00027**	0.000	0.2%	−0.01032	0.006	−7.5%	− 0.00008	0.000	0.2%	−0.0042	0.007	13.9%
**State**												
Tamil Nadu												
Jammu & Kashmir	0.00033	0.001	0.2%	−0.00015	0.000	−0.1%	−0.00118	0.001	3.9%	−0.0001	0.000	0.4%
Himachal Pradesh	0.00095*	0.000	0.7%	0.00010	0.000	0.1%	−0.00012	0.000	0.4%	−0.0002	0.000	0.6%
Punjab	0.00336*	0.002	2.5%	−0.00019	0.000	−0.1%	−0.00104	0.002	3.4%	−0.0003	0.000	1.0%
Chandigarh	0.00008*	0.000	0.1%	0.00000	0.000	0.0%	−0.00003	0.000	0.1%	0.0000	0.000	0.0%
Uttarakhand	0.00006	0.000	0.0%	0.00051	0.000	0.4%	0.00001	0.000	0.0%	−0.0003	0.000	0.8%
Haryana	0.00126**	0.000	0.9%	−0.00027	0.001	−0.2%	−0.00038	0.001	1.2%	0.0003	0.001	−1.1%
Delhi	0.00001	0.000	0.0%	−0.00026	0.001	−0.2%	−0.00001	0.000	0.0%	0.0002	0.001	−0.6%
Rajasthan	0.00068	0.000	0.5%	0.00025	0.002	0.2%	−0.00032	0.000	1.1%	0.0006	0.002	−2.1%
Uttar Pradesh	−0.00509*	0.002	−3.7%	0.01831***	0.006	13.4%	0.00480	0.005	−15.8%	−0.0050	0.005	16.5%
Bihar	−0.01576***	0.003	−11.5%	0.02031***	0.006	14.8%	0.01161	0.010	−38.3%	−0.01928**	0.006	63.5%
Arunachal Pradesh	0.00051**	0.000	0.4%	0.00011	0.000	0.1%	−0.00002	0.000	0.1%	0.0000	0.000	0.0%
Nagaland	0.00014	0.000	0.1%	0.00012	0.000	0.1%	0.00025	0.000	−0.8%	0.0000	0.000	0.0%
Manipur	0.00006	0.000	0.0%	0.00005	0.000	0.0%	−0.00018	0.000	0.6%	0.0000	0.000	0.1%
Mizoram	−0.00001	0.000	0.0%	−0.00004	0.000	0.0%	0.00001	0.000	0.0%	0.0000	0.000	−0.1%
Tripura	0.00034***	0.000	0.2%	0.00031*	0.000	0.2%	−0.00017	0.000	0.6%	−0.00039**	0.000	1.3%
Meghalaya	−0.00011***	0.000	0.1%	0.00021	0.000	0.2%	−0.00001	0.000	0.0%	−0.0001	0.000	0.2%
Assam	−0.00086***	0.000	−0.6%	0.00068	0.001	0.5%	0.00024	0.000	−0.8%	−0.0018	0.001	5.9%
West Bengal	0.00048	0.000	0.4%	−0.00079	0.003	−0.6%	−0.00017	0.000	0.5%	0.0007	0.003	−2.4%
Jharkhand	−0.00174	0.001	−1.3%	0.00279	0.001	2.0%	0.00047	0.001	−1.5%	−0.0018	0.001	6.0%
Odisha	−0.00415**	0.002	−3.0%	0.00746	0.003	5.4%	0.00199	0.002	−6.6%	−0.0037	0.003	12.3%
Chhattisgarh	−0.00120	0.002	−0.9%	0.00104	0.002	0.8%	−0.00047	0.001	1.6%	−0.0019	0.002	6.4%
Madhya Pradesh	−0.00013	0.000	0.1%	0.00518	0.003	3.8%	0.00018	0.000	−0.6%	−0.0055	0.003	18.1%
Gujarat	0.00002	0.000	0.0%	0.00223	0.002	1.6%	−0.00001	0.000	0.0%	−0.0030	0.002	10.0%
Daman & Diu	0.00001	0.000	0.0%	0.00000	0.000	0.0%	0.00000	0.000	0.0%	0.0000	0.000	0.0%
Dadra & Nagar Haveli	−0.00001	0.000	0.0%	0.00004	0.000	0.0%	0.00000	0.000	0.0%	0.0000	0.000	0.0%
Maharashtra	−0.00022	0.000	−0.2%	0.00019	0.004	0.1%	−0.00003	0.000	0.1%	−0.0009	0.004	3.0%
Andhra Pradesh	0.00483***	0.001	3.5%	0.00155	0.001	1.1%	−0.00045	0.001	1.5%	−0.0011	0.001	3.7%
Karnataka	0.00287	0.005	2.1%	0.00225	0.004	1.6%	0.00151	0.006	−5.0%	0.0006	0.003	−2.1%
Goa	0.00025***	0.000	0.2%	0.00002	0.000	0.0%	−0.00010	0.000	0.3%	0.0000	0.000	0.1%
Lakshadweep	0.00000	0.000	0.0%	0.00000	0.000	0.0%	0.00000	0.000	0.0%	0.0000	0.000	0.0%
Kerala	0.00013	0.000	0.1%	0.00046	0.001	0.3%	0.00009	0.000	−0.3%	−0.0001	0.001	0.2%
Puducherry	−0.00005	0.000	0.0%	0.00006	0.000	0.0%	0.00005	0.000	−0.2%	−0.0001	0.000	0.3%
Andaman & Nicobar Islands	0.00002***	0.000	0.0%	0.00000	0.000	0.0%	0.00000	0.000	0.0%	0.0000	0.000	0.0%
Telangana	0.00210***	0.001	1.5%	0.00091	0.001	0.7%	−0.00122	0.001	4.0%	−0.0012	0.001	4.1%
**Intercept**				0.06320	0.052	46.1%				0.11302*	0.050	−372.4%

*Note.*^a^divorced, separated, and deserted

^b^Activities of daily living

^c^Instrumental Activities of Daily Living (IADL)

^d^includes Sikh, Buddhist/neo-Buddhist, Jain, Parsi/Zoroastrian and others; and

^e^Other Backward Classes, HT-hypertension

*p < 0.05, **p < 0.01, ***p < 0.001

## Discussion

In order to achieve the global and national target of reducing premature deaths from HTN by 25% by 2025, there is a need to not only understand the SE disparities in the diagnosis and treatment of HTN but also to quantify the hidden burden of undiagnosed HTN and identify the individual and sub-group characteristics that are missed by the health system. In a country like India, where a large proportion of the population has barriers to access to health care, it is all the more essential to identify individuals who are missed out by the health system. Early diagnosis and prompt treatment are essential to minimize avoidable morbidity and mortality from CVDs and other diseases related to untreated HTN [9].

Our study shows that the overall prevalence of HTN among individuals aged 45 years and above in India is 47.5%. Of these, almost 40% are not aware of their hypertensive status, suggesting that SR measures underestimate the true prevalence of HTN, especially in low socio-economic groups. Similar to other studies, our study found that SR HTN increases with increase in age, level of education, and presence of co-morbidities such as diabetes, stroke, and arthritis, obesity and difficulties with ADLs [6, 13, 28, 29]. Our study also shows that socio-economic inequalities in SR HTN are highly concentrated among the educated and wealthy population in India. This finding is not surprising as education is a significant predictor of an individual’s health literacy [30, 31]. Higher HTN prevalence among higher SES can be explained by the higher prevalence of obesity, long working hours, sedentary lifestyle, and higher alcohol and salt intake [32]. Similar to other studies, we found that SR HTN was higher among urban areas, [11] whereas a higher proportion had undiagnosed HTN in rural areas. Our results imply that SR measures underestimate the prevalence of HTN and disproportionately affect the lower MPCE groups, thus exaggerating the health inequalities between the rich and the poor.

Consistent with other studies [33], our study found a positive concentration index for SR HTN (CI = 0.133), indicating that the richest group had a higher prevalence of HTN and that there was a substantial richest-poorest gap. On the other hand, we also found that SE inequality in undiagnosed HTN was significantly concentrated among the poorest group [8, 34–36].

Our decomposition analysis shows that the difference in the distribution of various SE and demographic characteristics plays an important role in explaining the poorest-richest gap in the case of SR HTN. Obesity and SR diabetes were key contributors to pro-rich inequality. Research from India suggests that older adults in the richest group have a greater prevalence of obesity than those in the poorest group [37]. In addition, they are more aware of their health status due to better health literacy and more access to health care than their poorest counterparts. This implies that reducing the richest-poorest gap in the prevalence of obesity can reduce the poorest-richest gap in the prevalence of SR HTN.

An interesting observation in our study was that inequality remains unexplained in the case of undiagnosed HTN. We hypothesize that host of unobserved factors are likely to explain the poor-rich inequality in the case of undiagnosed HTN, resulting in low MPCE quintiles having higher undiagnosed HTN. Some possible factors that could be contributing to this observation making the poor more vulnerable to HTN include lack of awareness, poverty, inability to buy health foods like fruits and vegetables, more intake of food with excess sodium, and financial barriers to access to health care services.

The lack of awareness can be attributed to a number of factors including reporting bias, issues around understanding and communication, and recall bias. There is evidence to suggest that poverty is an important risk factor for adverse health outcomes and that the poor are more likely to die prematurely from NCDs and CVDs [38–40]. Moreover, previous research has found that poorer population groups have limited health literacy, which is strongly associated with worse SR health status [41]. However, a major consideration in the Indian context is the financial barriers and lack of access to the health system. India is one of the few countries with over 60% out-of-pocket expenditure on health care [42]. As a result of the high out-of-pocket payments in India, NCDs are a significant contributor to poverty [43, 44]. For example, a study estimated that 1.4 million to 2 million Indians experienced catastrophic spending in 2004 as a result of costs of caring for cardiovascular disease and cancer [44]. With public spending on health making up only 30.6% of the total health expenditure, India’s public health care system suffers from chronic underinvestment [42]. This is reflected in the shortfall of human resources and other public heath infrastructure. For example, India has a low hospital beds to population ratio of 0.53 per 1000 population (in 2017) [45] and has an over 80% shortfall of specialists in community health centres [46]. Although home to 67% of India’s population, rural areas have only 33% of the total hospital beds [46]. Our findings confirm that a higher proportion of people who were poor and from the rural areas had undiagnosed HTN.

The fact that 40% of hypertensives in India are unaware of their condition and are missed out by the health care system has major policy implications. Existing government efforts of population-based screening and management of HTN at primary health care level based on early diagnosis and prompt treatment needs to be further strengthened. Particular emphasis should be given to outlier states as identified in the Figure 1. It is unfortunate that even those who access the health system for any medical condition are not screened for blood pressure. A recent study in India found that among those identified as having HTN around 23% of the undiagnosed hypertensives had contact with either a public or a private facility during the 1 year preceding the survey [47]. Addressing such missed opportunities can be a high-benefit-low-cost approach as hypertensive individuals can be diagnosed and treated at the earliest. There is evidence to suggest that routine opportunistic HTN screening at health facilities can significantly increase awareness of HTN in developing countries [48]. The Indian Medical Council (IMC) and other professional bodies must ensure that their members, both in the public and the private sectors, are sensitised to the need for opportunistic screening. Such opportunistic screening should supplement the existing government efforts of population-based screening. As HTN screening is not an end in itself, measures should be in place to ensure availability of free anti- hypertensive drugs and implementation of referral pathways for further management as appropriate since studies in India have shown low treatment and control rates even among those diagnosed [7].

The strength of our study is the use of a large, nationally-representative sample using both decomposition analysis and concentration indices to analyse the determinants of SE inequalities among older adults. To the best of our knowledge, this is one of the first studies to compare and explain SE inequalities among the SR and undiagnosed hypertensives. All the limitations of a cross-sectional survey apply to our study, including recall bias, blood pressure measurement bias, low reliability of SR data, and inability to draw any causal inferences. In addition, as the study excluded adults between 30 and 45 years, the generalisability of study finding may be restricted to adults over the age of 45 years only.

## Conclusion

The present study showed that a large proportion of the hypertensive cases remained undiagnosed in India, especially among the low socio-economic groups. Furthermore, SR measures of hypertension generally underestimate the true prevalence of HTN. Therefore, awareness about routine blood pressure level check-ups and follow-ups is needed. In order to meet the national and global NCD targets, India has to strengthen its ailing public health sector. The chronically underfunded public health system, focused on communicable diseases and activities related to maternal and child health, needs to be re-oriented to meet the challenges and the growing burden of NCDs and HTN. Given the scarcity of resources, the importance of opportunistic screening for HTN by both public and private health facilities cannot be overemphasized. Focussing on the hidden burden of undiagnosed HTN among individuals and sub-groups missed by the health system and adopting the primary health care approach would not only ensure early diagnosis and prompt treatment of HTN but also reduce SE inequalities.

## Supplementary Information

**Additional file 1: Table S1.** Characteristics of the study participants, LASI Wave 1, 2017–18. **Table S2.** Concentration Index (CI) of self-reported, undiagnosed, and overall hypertension by various background characteristics, India, LASI, 2017–18. **Figure S1.** Scatter plots. (A) Gap in self-reported between the individuals in poorest and richest groups (B) Gap in self-reported between the individuals in poorest and richest groups, LASI, 2017–18.

## Data Availability

The study used secondary data which is available in the private database and accessible on reasonable request at https://www.iipsindia.ac.in/content/lasi-wave-i.

## Abbreviations

ADL: Activities of Daily Living
BMI: Body Mass Index
CIs: Concentration indices
CVDs: Cardiovascular diseases
DALYs: Disability Adjusted Life Years
DBP: Diastolic Blood Pressure
LASI: Longitudinal Aging Study in India
HTN: Hypertension
MPCE: Monthly per capita expenditure
NCDs: Non-communicable diseases
SBP: Systolic Blood Pressure
SES: Socio-Economic Status
SR: Self-reported
SR HTN: Self-reported hypertension
