# Supplementary material for: Comparing socio-economic inequalities in self-reported and undiagnosed hypertension among adults 45 years and over in India: what explains these inequalities?
Source: Int J Equity Health. 2023 Feb 2;22:26. doi: 10.1186/s12939-023-01833-6 (PMC9893593; doi:10.1186/s12939-023-01833-6)
Supplement: Supplementary file 1 — Additional file 1: Table S1. Characteristics of the study participants, LASI Wave 1, 2017–18. Table S2. Concentration Index (CI) of self-reported, undiagnosed, and overall hypertension by various background characteristics, India, LASI, 2017–18. Figure S1. Scatter plots. (A) Gap in self-reported between the individuals in poorest and richest groups (B) Gap in self-reported between the individuals in poorest and richest groups, LASI, 2017–18. [file 12939_2023_1833_MOESM1_ESM.docx]

| **Table S1. Characteristics of the study participants, LASI Wave 1, 2017-18** | | | | | | |
| --- | --- | --- | --- | --- | --- | --- |
|  | **Overall** | | **Men** | | **Women** | |
|  | **N** | **%** | **N** | **%** | **N** | **%** |
| **Individual factors** | | | | | | |
| **Age groups** |  |  |  |  |  |  |
| 45-54 | 24,094 | 36.7 | 10,913 | 35.8 | 13,181 | 37.6 |
| 55-64 | 20,136 | 30.7 | 9,106 | 29.9 | 11,030 | 31.4 |
| 65-74 | 14,583 | 22.2 | 7,218 | 23.7 | 7,365 | 21.0 |
| 75+ | 6,749 | 10.3 | 3,242 | 10.6 | 3,507 | 10.0 |
| **Education level** |  |  |  |  |  |  |
| No education | 30,818 | 47.0 | 9,484 | 31.1 | 21,334 | 60.8 |
| Primary | 16,096 | 24.6 | 8,781 | 28.8 | 7,315 | 20.9 |
| Secondary | 12,126 | 18.5 | 7,683 | 25.2 | 4,443 | 12.7 |
| Higher | 6,519 | 9.9 | 4,531 | 14.9 | 1,988 | 5.7 |
| **Working Status** |  |  |  |  |  |  |
| Never worked | 17,986 | 27.4 | 1,250 | 4.1 | 16,736 | 47.7 |
| Currently working | 30,177 | 46.0 | 19,788 | 64.9 | 10,389 | 29.6 |
| Not currently working | 17,383 | 26.5 | 9,430 | 31.0 | 7,953 | 22.7 |
| **Marital Status** |  |  |  |  |  |  |
| Currently married | 48,769 | 74.4 | 26,711 | 87.6 | 22,058 | 62.9 |
| Widowed | 14,592 | 22.3 | 2,773 | 9.1 | 11,819 | 33.7 |
| D/S/D/Others^1^ | 2,198 | 3.4 | 994 | 3.3 | 1,204 | 3.4 |
| **Morbidities** | | | | | | |
| **Diabetes** |  |  |  |  |  |  |
| No | 56,952 | 87.1 | 26,327 | 86.7 | 30,625 | 87.5 |
| Yes | 8,429 | 12.9 | 4,045 | 13.3 | 4,384 | 12.5 |
| **Stroke** |  |  |  |  |  |  |
| No | 64,195 | 98.2 | 29,660 | 97.6 | 34,535 | 98.6 |
| Yes | 1,195 | 1.8 | 720 | 2.4 | 475 | 1.4 |
| **Arthritis** |  |  |  |  |  |  |
| No | 60,065 | 91.9 | 28,454 | 93.7 | 31,611 | 90.3 |
| Yes | 5,327 | 8.1 | 1,926 | 6.3 | 3,401 | 9.7 |
| **Difficulty in ADL^2^** |  |  |  |  |  |  |
| No | 55,740 | 85.4 | 26,632 | 87.8 | 29,108 | 83.3 |
| Yes | 9,541 | 14.6 | 3,689 | 12.2 | 5,852 | 16.7 |
| **Difficulty in IADL^3^** |  |  |  |  |  |  |
| No | 43,524 | 66.7 | 22,820 | 75.3 | 20,704 | 59.3 |
| Yes | 21,698 | 33.3 | 7,481 | 24.7 | 14,217 | 40.7 |
| **BMI categories** |  |  |  |  |  |  |
| Normal | 30,872 | 52.3 | 15,557 | 56.9 | 15,315 | 48.3 |
| Underweight | 10,963 | 18.6 | 5,273 | 19.3 | 5,690 | 17.9 |
| Overweight/obese | 17,238 | 29.2 | 6,513 | 23.8 | 10,725 | 33.8 |
| **Lifestyle factors** | | | | | | |
| **Moderate activities** |  |  |  |  |  |  |
| Inactive | 24,886 | 38.3 | 14,301 | 47.4 | 10,585 | 30.4 |
| Active | 40,115 | 61.7 | 15,866 | 52.6 | 24,249 | 69.6 |
| **Vigorous activities** |  |  |  |  |  |  |
| Inactive | 44,883 | 69.1 | 18,077 | 59.9 | 26,806 | 77.0 |
| Active | 20,110 | 30.9 | 12,084 | 40.1 | 8,026 | 23.0 |
| **Smoking status** |  |  |  |  |  |  |
| Never | 53,067 | 81.7 | 19,822 | 65.7 | 33,245 | 95.4 |
| Former | 2,997 | 4.6 | 2,584 | 8.6 | 413 | 1.2 |
| Current | 8,922 | 13.7 | 7,749 | 25.7 | 1,173 | 3.4 |
| **Chewing tobacco status** |  |  |  |  |  |  |
| Never | 50,863 | 78.3 | 21,654 | 71.8 | 29,209 | 83.9 |
| Former | 1,662 | 2.6 | 1,077 | 3.6 | 585 | 1.7 |
| Current | 12,461 | 19.2 | 7,425 | 24.6 | 5,036 | 14.5 |
| **Alcohol use** |  |  |  |  |  |  |
| No | 53,290 | 82.0 | 19,883 | 65.9 | 33,407 | 95.9 |
| Yes | 11,718 | 18.0 | 10,282 | 34.1 | 1436 | 4.1 |
| **Household factors** | | | | | | |
| **MPCE quintile** |  |  |  |  |  |  |
| Poorest | 12,941 | 19.7 | 5,965 | 19.6 | 6,976 | 19.9 |
| Poorer | 13,190 | 20.1 | 6,086 | 20.0 | 7,104 | 20.2 |
| Middle | 13,163 | 20.1 | 6,105 | 20.0 | 7,058 | 20.1 |
| Richer | 13,210 | 20.1 | 6,206 | 20.4 | 7,004 | 20.0 |
| Richest | 13,058 | 19.9 | 6,117 | 20.1 | 6,941 | 19.8 |
| **Religion** |  |  |  |  |  |  |
| Hindu | 48,099 | 73.4 | 22,450 | 73.7 | 25,649 | 73.1 |
| Muslim | 7,803 | 11.9 | 3,549 | 11.6 | 4,254 | 12.1 |
| Christian | 6,536 | 10.0 | 3,002 | 9.8 | 3,534 | 10.1 |
| Others^4^ | 3,120 | 4.8 | 1,478 | 4.8 | 1,642 | 4.7 |
| **Caste** |  |  |  |  |  |  |
| Scheduled Caste | 10,959 | 17.3 | 5,049 | 17.2 | 5,910 | 17.5 |
| Scheduled Tribe | 11,365 | 18.0 | 5,256 | 17.9 | 6,109 | 18.1 |
| OBC^5^ | 24,629 | 39.0 | 11,521 | 39.2 | 13,108 | 38.8 |
| Others | 16,279 | 25.7 | 7,597 | 25.8 | 8,682 | 25.7 |
| **Place of Residence** |  |  |  |  |  |  |
| Rural | 42,424 | 64.7 | 19,889 | 65.3 | 22,535 | 64.2 |
| Urban | 23,138 | 35.3 | 10,590 | 34.7 | 12,548 | 35.8 |
| **Region** |  |  |  |  |  |  |
| North | 11,966 | 18.3 | 5,552 | 18.2 | 6,414 | 18.3 |
| Central | 8,907 | 13.6 | 4,304 | 14.1 | 4,603 | 13.1 |
| East | 11,580 | 17.7 | 5,469 | 17.9 | 6,111 | 17.4 |
| Northeast | 8,513 | 13.0 | 4,077 | 13.4 | 4,436 | 12.6 |
| West | 8,894 | 13.6 | 4,041 | 13.3 | 4,853 | 13.8 |
| South | 15,702 | 23.9 | 7,036 | 23.1 | 8,666 | 24.7 |
|  |  |  |  |  |  |  |
| **Total** | **65,562** | **100** | **30,479** | **100** | **35,083** | **100** |
| *Note.* ^1^divorced, separated, and deserted; ^2^Activities of daily living; ^3^Instrumental Activities of Daily Living (IADL); ^4^includes Sikh, Buddhist/neo-Buddhist, Jain, Parsi/Zoroastrian and others; and ^5^Other Backward Classes. | | | | | | |

| **Table S2. Concentration Index (CI) of self-reported, undiagnosed, and overall hypertension by various background characteristics, India, LASI, 2017-18** | | | | | | | | | | | | |
| --- | --- | --- | --- | --- | --- | --- | --- | --- | --- | --- | --- | --- |
|  | **Self-reported HT** | | | | **Undiagnosed HT** | | | | **Overall HT** | | | |
|  | **Poorest (%)** | **Richest (%)** | **CI** | **p-value** | **Poorest (%)** | **Richest (%)** | **CI** | **p-value** | **Poorest (%)** | **Richest (%)** | **CI** | **p-value** |
| **Overall** | **21.0** | **35.9** | **0.133** | **0.000** | **19.6** | **16.2** | **-0.047** | **0.000** | **40.6** | **52.1** | **0.062** | **0.000** |
| **Individual factors** |  |  |  |  |  |  |  |  |  |  |  |  |
| **Age groups** |  |  |  |  |  |  |  |  |  |  |  |  |
| 45-54 | 13.9 | 29.2 | 0.177 | 0.000 | 16.6 | 16.8 | 0.003 | 0.777 | 30.5 | 46.1 | 0.101 | 0.000 |
| 55-64 | 20.8 | 36.4 | 0.139 | 0.000 | 20.8 | 13.8 | -0.100 | 0.000 | 41.6 | 50.2 | 0.047 | 0.000 |
| 65-74 | 26.5 | 42.7 | 0.119 | 0.000 | 21.2 | 17.4 | -0.047 | 0.000 | 47.6 | 60.1 | 0.058 | 0.000 |
| 75+ | 29.7 | 45.2 | 0.104 | 0.000 | 21.4 | 17.9 | -0.042 | 0.028 | 51.1 | 63.1 | 0.052 | 0.000 |
| **Sex** |  |  |  |  |  |  |  |  |  |  |  |  |
| Men | 17.9 | 30.9 | 0.135 | 0.000 | 19.5 | 19.4 | -0.001 | 0.938 | 37.4 | 50.4 | 0.074 | 0.000 |
| Women | 23.5 | 40.3 | 0.134 | 0.000 | 19.6 | 13.3 | -0.094 | 0.000 | 43.2 | 53.6 | 0.054 | 0.000 |
| **Education level** |  |  |  |  |  |  |  |  |  |  |  |  |
| No education | 19.1 | 31.9 | 0.122 | 0.000 | 20.1 | 16.5 | -0.043 | 0.000 | 39.2 | 48.4 | 0.048 | 0.000 |
| Primary | 24.1 | 38.1 | 0.114 | 0.000 | 20.8 | 16.6 | -0.055 | 0.000 | 44.9 | 54.7 | 0.049 | 0.000 |
| Secondary | 25.6 | 37.7 | 0.088 | 0.000 | 14.9 | 15.4 | 0.008 | 0.621 | 40.5 | 53.1 | 0.063 | 0.000 |
| Higher | 20.2 | 39.0 | 0.081 | 0.000 | 19.1 | 15.9 | -0.029 | 0.063 | 39.3 | 54.9 | 0.046 | 0.000 |
| **Working Status** |  |  |  |  |  |  |  |  |  |  |  |  |
| Never worked | 28.6 | 46.2 | 0.117 | 0.000 | 16.2 | 11.8 | -0.079 | 0.000 | 44.7 | 58.0 | 0.064 | 0.000 |
| Currently working | 13.9 | 24.6 | 0.142 | 0.000 | 20.2 | 19.9 | -0.003 | 0.704 | 34.1 | 44.5 | 0.067 | 0.000 |
| Not currently working | 26.6 | 43.2 | 0.121 | 0.000 | 21.4 | 14.8 | -0.088 | 0.000 | 47.9 | 58.1 | 0.048 | 0.000 |
| **Marital Status** |  |  |  |  |  |  |  |  |  |  |  |  |
| Currently married | 19.0 | 33.8 | 0.141 | 0.000 | 17.9 | 16.0 | -0.028 | 0.001 | 37.0 | 49.9 | 0.074 | 0.000 |
| Widowed | 26.7 | 44.5 | 0.127 | 0.000 | 23.7 | 17.2 | -0.072 | 0.000 | 50.3 | 61.8 | 0.050 | 0.000 |
| D/S/D/Others^1^ | 16.5 | 32.7 | 0.169 | 0.000 | 21.8 | 11.5 | -0.147 | 0.000 | 38.3 | 44.1 | 0.035 | 0.074 |
| **Morbidities** |  |  |  |  |  |  |  |  |  |  |  |  |
| **Diabetes** |  |  |  |  |  |  |  |  |  |  |  |  |
| No | 17.9 | 27.6 | 0.108 | 0.000 | 20.3 | 18.1 | -0.028 | 0.000 | 38.2 | 45.6 | 0.044 | 0.000 |
| Yes | 55.2 | 70.5 | 0.052 | 0.000 | 12.5 | 8.6 | -0.088 | 0.000 | 67.7 | 79.1 | 0.034 | 0.000 |
| **Stroke** |  |  |  |  |  |  |  |  |  |  |  |  |
| No | 20.7 | 35.1 | 0.131 | 0.000 | 19.7 | 16.4 | -0.044 | 0.000 | 40.3 | 51.6 | 0.061 | 0.000 |
| Yes | 44.8 | 71.0 | 0.107 | 0.000 | 17.3 | 6.3 | -0.247 | 0.000 | 62.1 | 77.3 | 0.052 | 0.000 |
| **Arthritis** |  |  |  |  |  |  |  |  |  |  |  |  |
| No | 20.4 | 34.1 | 0.128 | 0.000 | 20.0 | 17.0 | -0.040 | 0.000 | 40.4 | 51.1 | 0.059 | 0.000 |
| Yes | 29.3 | 50.7 | 0.126 | 0.000 | 14.6 | 9.7 | -0.101 | 0.001 | 43.9 | 60.4 | 0.076 | 0.000 |
| **Difficulty in ADL^2^** |  |  |  |  |  |  |  |  |  |  |  |  |
| No | 18.7 | 34.3 | 0.150 | 0.000 | 19.7 | 16.4 | -0.046 | 0.000 | 38.4 | 50.7 | 0.069 | 0.000 |
| Yes | 31.9 | 45.0 | 0.086 | 0.000 | 19.2 | 15.7 | -0.048 | 0.005 | 51.1 | 60.7 | 0.043 | 0.000 |
| **Difficulty in IADL^3^** |  |  |  |  |  |  |  |  |  |  |  |  |
| No | 18.5 | 32.8 | 0.140 | 0.000 | 19.1 | 17.0 | -0.029 | 0.000 | 37.6 | 49.8 | 0.070 | 0.000 |
| Yes | 24.8 | 42.6 | 0.135 | 0.000 | 20.4 | 14.8 | -0.076 | 0.000 | 45.2 | 57.4 | 0.059 | 0.000 |
| **Lifestyle factors** |  |  |  |  |  |  |  |  |  |  |  |  |
| **BMI categories** |  |  |  |  |  |  |  |  |  |  |  |  |
| Normal | 20.3 | 28.2 | 0.082 | 0.000 | 21.7 | 18.1 | -0.043 | 0.000 | 42.0 | 46.3 | 0.024 | 0.000 |
| Underweight | 13.3 | 21.0 | 0.096 | 0.000 | 19.4 | 14.8 | -0.048 | 0.001 | 32.7 | 35.8 | 0.018 | 0.060 |
| Overweight/obese | 33.5 | 45.8 | 0.068 | 0.000 | 24.2 | 20.0 | -0.045 | 0.000 | 57.7 | 65.8 | 0.030 | 0.000 |
| **Moderate activities** |  |  |  |  |  |  |  |  |  |  |  |  |
| Inactive | 23.3 | 37.7 | 0.119 | 0.000 | 19.8 | 16.6 | -0.043 | 0.000 | 43.1 | 54.3 | 0.058 | 0.000 |
| Active | 19.5 | 35.1 | 0.145 | 0.000 | 19.7 | 16.2 | -0.048 | 0.000 | 39.2 | 51.3 | 0.067 | 0.000 |
| **Vigorous activities** |  |  |  |  |  |  |  |  |  |  |  |  |
| Inactive | 24.2 | 39.0 | 0.118 | 0.000 | 19.6 | 14.9 | -0.067 | 0.000 | 43.8 | 53.9 | 0.052 | 0.000 |
| Active | 13.8 | 29.5 | 0.186 | 0.000 | 20.1 | 19.6 | -0.006 | 0.596 | 33.8 | 49.1 | 0.093 | 0.000 |
| **Smoking status** |  |  |  |  |  |  |  |  |  |  |  |  |
| Never | 21.4 | 38.0 | 0.142 | 0.000 | 20.1 | 16.1 | -0.055 | 0.000 | 41.5 | 54.1 | 0.066 | 0.000 |
| Former | 27.4 | 33.3 | 0.049 | 0.026 | 14.9 | 20.4 | 0.078 | 0.012 | 42.3 | 53.7 | 0.060 | 0.000 |
| Current | 16.1 | 23.2 | 0.090 | 0.000 | 18.4 | 16.8 | -0.022 | 0.236 | 34.5 | 40.0 | 0.036 | 0.001 |
| **Chewing tobacco Status** |  |  |  |  |  |  |  |  |  |  |  |  |
| Never | 22.3 | 37.6 | 0.129 | 0.000 | 19.1 | 15.5 | -0.051 | 0.000 | 41.3 | 53.1 | 0.063 | 0.000 |
| Former | 29.5 | 37.6 | 0.060 | 0.025 | 17.7 | 21.0 | 0.043 | 0.276 | 47.2 | 58.6 | 0.054 | 0.003 |
| Current | 15.7 | 26.6 | 0.125 | 0.000 | 22.0 | 20.5 | -0.015 | 0.244 | 37.7 | 47.1 | 0.052 | 0.000 |
| **Alcohol use** |  |  |  |  |  |  |  |  |  |  |  |  |
| No | 22.0 | 37.1 | 0.129 | 0.000 | 18.9 | 15.3 | -0.052 | 0.000 | 40.9 | 52.5 | 0.062 | 0.000 |
| Yes | 15.2 | 29.2 | 0.162 | 0.000 | 23.9 | 22.9 | -0.011 | 0.408 | 39.1 | 52.0 | 0.071 | 0.000 |
| **Household factors** |  |  |  |  |  |  |  |  |  |  |  |  |
| **Religion** |  |  |  |  |  |  |  |  |  |  |  |  |
| Hindu | 19.7 | 33.5 | 0.132 | 0.000 | 19.6 | 16.5 | -0.043 | 0.000 | 39.3 | 50.0 | 0.060 | 0.000 |
| Muslim | 30.0 | 51.0 | 0.131 | 0.000 | 18.7 | 12.8 | -0.090 | 0.000 | 48.8 | 63.8 | 0.067 | 0.000 |
| Christian | 19.1 | 34.8 | 0.149 | 0.000 | 22.3 | 16.8 | -0.067 | 0.000 | 41.3 | 51.6 | 0.055 | 0.000 |
| Others^4^ | 24.2 | 42.0 | 0.107 | 0.000 | 17.9 | 17.7 | -0.002 | 0.939 | 42.1 | 59.7 | 0.071 | 0.000 |
| **Caste** |  |  |  |  |  |  |  |  |  |  |  |  |
| Scheduled Caste | 19.6 | 32.2 | 0.117 | 0.000 | 17.8 | 17.5 | -0.004 | 0.802 | 37.4 | 49.7 | 0.066 | 0.000 |
| Scheduled Tribe | 9.0 | 23.3 | 0.214 | 0.000 | 23.4 | 18.4 | -0.042 | 0.000 | 32.4 | 41.8 | 0.050 | 0.000 |
| OBC^5^ | 22.3 | 36.6 | 0.123 | 0.000 | 20.4 | 16.2 | -0.057 | 0.000 | 42.6 | 52.7 | 0.053 | 0.000 |
| Others | 27.6 | 38.1 | 0.070 | 0.000 | 16.8 | 15.3 | -0.022 | 0.110 | 44.5 | 53.5 | 0.041 | 0.000 |
| **Place of Residence** |  |  |  |  |  |  |  |  |  |  |  |  |
| Rural | 16.1 | 30.1 | 0.155 | 0.000 | 19.8 | 17.2 | -0.035 | 0.000 | 35.9 | 47.3 | 0.069 | 0.000 |
| Urban | 31.3 | 46.9 | 0.100 | 0.000 | 19.1 | 14.2 | -0.073 | 0.000 | 50.4 | 61.1 | 0.048 | 0.000 |
| **Region** |  |  |  |  |  |  |  |  |  |  |  |  |
| North | 25.6 | 36.9 | 0.080 | 0.000 | 15.9 | 16.3 | 0.005 | 0.748 | 41.5 | 53.2 | 0.056 | 0.000 |
| Central | 14.1 | 28.4 | 0.170 | 0.000 | 19.4 | 14.5 | -0.063 | 0.000 | 33.5 | 42.8 | 0.057 | 0.000 |
| East | 20.1 | 36.3 | 0.141 | 0.000 | 19.2 | 13.9 | -0.067 | 0.000 | 39.3 | 50.2 | 0.056 | 0.000 |
| Northeast | 23.7 | 32.6 | 0.079 | 0.000 | 24.4 | 16.5 | -0.095 | 0.000 | 48.2 | 49.1 | 0.005 | 0.567 |
| West | 25.1 | 33.6 | 0.072 | 0.000 | 20.6 | 18.0 | -0.033 | 0.053 | 45.8 | 51.6 | 0.030 | 0.001 |
| South | 25.7 | 40.1 | 0.100 | 0.000 | 20.1 | 17.1 | -0.040 | 0.002 | 45.8 | 57.2 | 0.052 | 0.000 |
| *Note.* ^1^divorced, separated, and deserted; ^2^Activities of daily living; ^3^Instrumental Activities of Daily Living (IADL); ^4^includes Sikh, Buddhist/neo-Buddhist, Jain, Parsi/Zoroastrian and others; and ^5^Other Backward Classes, HT-hypertension. | | | | | | | | | | | | |

Figure S1. Scatter plots. (A) Gap in self-reported between the individuals in poorest and richest groups (B) Gap in self-reported between the individuals in poorest and richest groups, LASI, 2017-18.


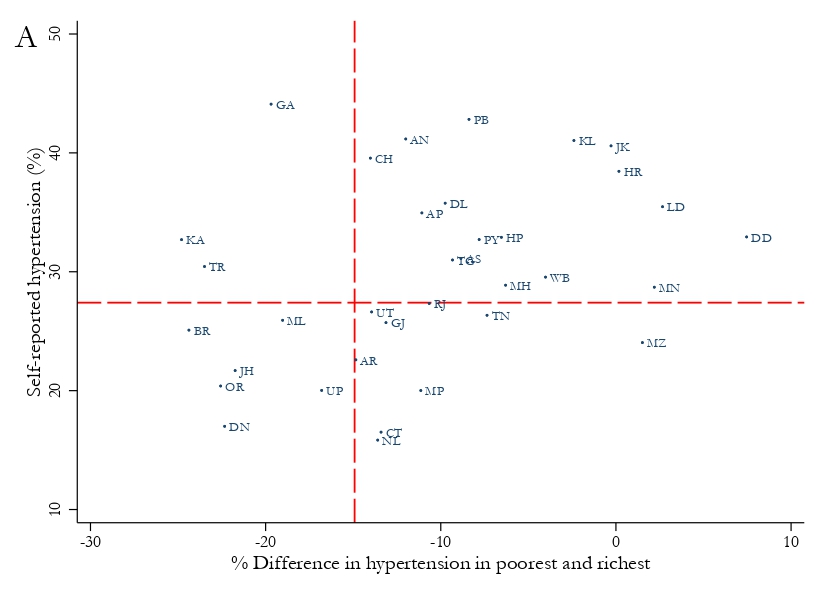


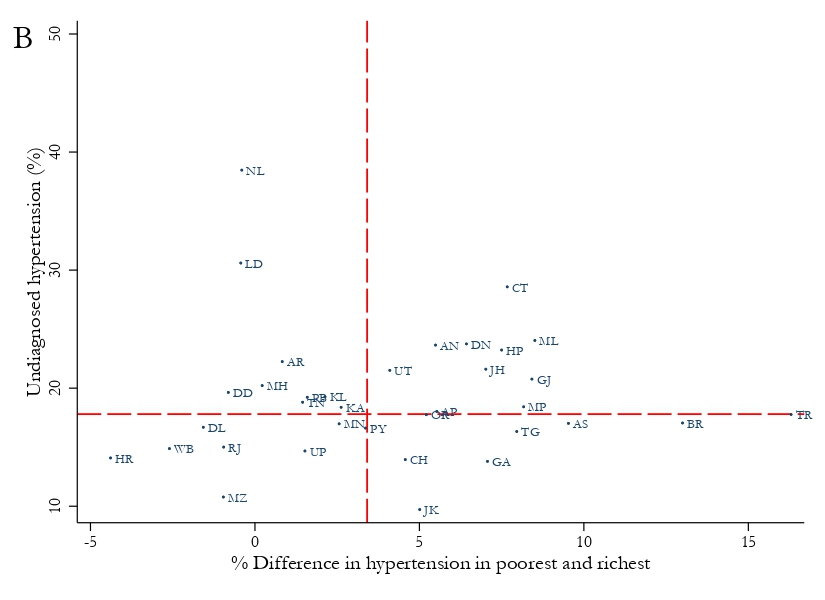


Note. Jammu & Kashmir (JK), Himachal Pradesh (HP), Punjab (PB), Chandigarh (CH), Uttarakhand (UT), Haryana (HR), Delhi (DL), Rajasthan (RJ), Uttar Pradesh (UP), Bihar (BR), Arunachal Pradesh (AR), Nagaland (NL), Manipur (MN), Mizoram (MZ), Tripura (TR), Meghalaya (ML), Assam (AS), West Bengal (WB), Jharkhand (JH), Odisha (OR), Chhattisgarh (CT), Madhya Pradesh (MP), Gujarat (GJ), Daman & Diu (DD), Dadra & Nagar Haveli (DN), Maharashtra (MH), Andhra Pradesh (AP), Karnataka (KA), Goa (GA), Lakshadweep (LD), Kerala (KL), Tamil Nadu (TN), Puducherry (PY), Andaman & Nicobar Island (AN), and Telangana (TG).

**Sample design of the study**

LASI is a multipurpose nationally representative longitudinal survey that provides information on demographics, cognition, mental and physical health, and social networks of the aging population in India. It is India’s first aging study with a sample size of more than 72000 individuals aged 45 years and older across all states and union territories. Wave 1 for LASI was conducted during the years 2017 to 2019. In LASI wave 1, the sample selection is based on a multistage stratified probability cluster sampling design. Within each state, LASI used a three-stage sampling design in rural areas and a four-stage sampling design in urban areas. The selection of PSUs (primary sampling units) and SSUs (secondary sampling units) was derived from the 2011 Census (Census of India, 2011). Selected SSUs underwent the process of mapping and listing to provide the updated SSU-wise household sampling frames which was used to select the LASI households randomly. Further details of sampling design, survey instruments, and data collection procedures are provided elsewhere (IIPS et al., 2020).

**Calculation of concentration index**

The concentration index (CI) quantifies the degree of socioeconomic-related inequality in the outcome variable. It is defined as the twice the area between the curve of equality (the 45-degree line) and concentration curve and its value lie between -1 and +1. The CI with value of zero means there is no socioeconomic inequality in the variable of interest. The negative value of CI suggests that the concentration curve lies above the line of equality indicates that there is disproportionate concentration of outcome variable in among poor. On the other hand, positive value of the CI suggests that concentration lies below the line of equality and indicates the disproportionate concentration of the outcome variable among the “rich”. The concentration index in mathematical terms is given as:

$$C=\frac{2}{\mu}cov(h,r)$$

Where, h is the health outcome variable, $\mu$ is the mean of the health outcome variable, and r is the rank of the individual in the wealth distribution.

**Decomposing the inequality**

We used logit-based multivariate decomposition analysis for non-linear outcome variables “self-reported hypertension” and “undiagnosed hypertension”, to assess the contribution of various background characteristics in the observed difference in these prevalences between the two groups (richest and poorest). Please refer below for further details.

Powers, D., Yoshioka, H., & Yun, M. (2011). Mvdcmp: Multivariate decomposition for nonlinear response models. Stata Journal, 11(4), 556–576.
